# The Treatment Needs and Experiences of Pedohebephiles: A Systematic Review

**DOI:** 10.1007/s10508-024-02943-0

**Published:** 2024-07-15

**Authors:** Agatha Chronos, Sara Jahnke, Nicholas Blagden

**Affiliations:** 1 Department of Health Promotion and Development, University of Bergen, Årstadveien 17, 5009 Bergen, Norway; 2 School for Law and Social Sciences, University of Derby, Derby, UK

**Keywords:** Pedohebephilia, Treatment, Pedophilia, Ephebophilia, Teleiophilia, DSM-5

## Abstract

People with a sexual interest in children face significant barriers to seeking and receiving mental health treatment. This review aims to bridge the gap between the treatment needs and experiences of pedohebephiles, and the services aiming to support them. Reviewers screened 1705 database hits and extracted findings from 22 qualitative, 15 quantitative, and 3 mixed-method studies on the treatment needs and experiences of pedohebephiles. Research suggests that this population experiences significant levels of distress, depression, and anxiety related to their sexual interest. Many individuals belonging to this population would seek (median = 42.3%), or have sought (median = 46.5%), treatment to cope with their sexual interest or with potential related mental health repercussions. Their experiences in treatment have been mixed, with some reporting positive experiences with empathic therapists and others reporting rejection. Most frequently, pedohebephiles report fear of exposure and rejection as barriers to seeking treatment, in addition to fear of the legal repercussions. The current study is the first to summarize and discuss previous findings on the treatment needs and experiences of pedohebephiles. The findings indicate that the treatment needs of pedohebephiles often remain unaddressed. Suggestions to increase the fit between treatment services and the needs of pedohebephiles are put forward.

**Supplementary Information:**

The online version contains supplementary material available at 10.1007/s10508-024-02943-0.

## Introduction

Traditionally, treatment for people with a sexual interest in children has occurred in forensic settings after a sexual offense. Recent years have witnessed a rise in treatment options for this particular group in community settings, often with the primary goal of preventing sexual offending (Beier et al., 2009, 2021). Although our understanding of the needs and treatment experiences for this population is limited, there is a growing body of research exploring various aspects of sexual attraction to children and treatment from their own perspective (Cacciatori, 2017; Lievesley et al., 2023). A better understanding of their treatment needs, experiences, and barriers is essential for providing effective and tailored interventions. Therefore, the present article will systematically review the extant literature to support ongoing efforts to provide appropriate support and mental health services for people with a sexual interest in children.

The terminology used to describe sexual attraction to children is multitudinous, controversial, and widely disputed (Jahnke et al., 2022a). While the most commonplace term is “pedophilia,” some have advocated against its use due to the stigmatizing, negative connotation that a pedophile is also a child sex offender, but also because it is not an all-encompassing term for sexual attraction to children (Parr & Pearson, 2019). Pedophilia only accounts for the attraction to prepubescents (including nepiophilia, the attraction to young children and infants); however, hebephilia includes the attraction to pubescent children (Seto, 2017). Investigating pedohebephilia, which has been found to comprise a set of related sexual interests (Stephens et al., 2017), allows for a more comprehensive investigation of sexual interest in children. Similarly, the term “minor-attracted person” is problematic for the purpose of this review because although it has been proposed as a less stigmatizing term (Chamandy, 2020), the identification of an individual as a minor is a variable legal standard, and often includes post-pubescents whose secondary sexual characteristics would categorize them as the objects of ephebophilic (sexual interest in mid-to-late adolescents) or even teleiophilic (sexual interest in adults) attractions. It is questionnable whether using person-first or identity-first language, such as “person with pedohebephilia” or “pedohebephilic person” represents a viable alternative, as this term may be perceived as more stigmatizing than the term “pedohebephile” (Jahnke et al., 2022a). Therefore, the terms “pedohebephile” and “pedohebephilia”—defined as primary or exclusive attraction to pubescent and/or prepubescent children—will be utilized throughout the current review, in addition to—and synonymous with—“people with a sexual interest in children.”

It is difficult to ascertain the true prevalence of pedohebephilia in the general population due to the stigma associated with reporting a sexual interest in children. Researchers found that in all-male samples, approximately 4.1% (Dombert et al., 2016) expressed pedohebephilic attraction. Others found that hebephilic interest was present in 16.8% of men and 1.4% of women, and pedophilic interest was present in 2.3% of men and 0.4% of women (Bártová et al., 2021). Note that estimate rates depend on whether pedophilia or hebephilia is defined as preferential or any sexual interest in children. People with a sexual interest in children are often subjected to social stigma and ostracism due to the common misperception that sexual interest in children is synonymous with sexual offending against children (Walker, 2017). When discussing this population, it is important to differentiate between action and attraction. While all pedohebephiles experience sexual attraction towards pubescent and/or prepubescent children, some of them perpetrate sexual offenses against children, and others refrain from ever doing so. Although pedohebephilic interest is a risk factor for child sexual abuse (Dombert et al., 2016), it does not mean that pedohebephiles are doomed to commit child sex offences. In fact, there are large communities of people with a sexual interest in children that are aware of their interests and take steps to avoid acting on them (Cacciatori, 2017).

Pedohebephiles that have perpetrated sexual offenses are more likely to have antisocial traits, and to find themselves facing mandated treatment which could lead to very different treatment experiences (Seto, 2018). Therefore, it stands to reason that the needs and perspectives of pedohebephiles stemming from community settings would differ from those in forensic or clinical settings. Furthermore, many practitioners are not aware of their needs and/or may have different goals for treatment than their clients (Lievesley et al., 2023). There are also concerns that clients’ needs are not being met in treatment or that they face significant barriers when seeking to access treatment in community settings (Walker, 2017). Previous systematic reviews have focused on community intervention programs for child sex offenders and their impact on recidivism rates (Barros et al., 2022; Långström et al., 2013), on the efficacy of specific interventions for reducing pedohebephilic or pedophilic arousal (McPhail & Olver, 2020), on the effects of stigmatization on people with a sexual interest in children (Jahnke & Hoyer, 2013; Montgomery-Ferrer, 2023), on the prevalence and correlates of pedohebephilia (Savoie et al., 2021), or on lay persons’ beliefs and myths regarding sexual interest in children (Glina et al., 2022). To the best of our knowledge, no previous review has synthesized the evidence base on the treatment needs and experiences of pedohebephiles. A systematic review is pertinent for investigating what pedohebephiles want when it comes to treatment, and the factors that may impede them from accessing it. This knowledge is needed to reach individuals in need of support and develop treatment plans that are tailored to their needs so that they can lead fulfilling lives and manage their attractions.

In this systematic review, we aim to shed some light on treatment needs and experiences from pedohebephiles’ perspectives. We aim to explore their likelihood of seeking or having sought treatment, motives for which they would seek treatment, their opinions and attitudes regarding treatment, in addition to previous experiences in treatment and factors they consider to facilitate or impede their seeking treatment. The review will include findings pertaining to forensic, clinical, community, and mixed settings. The outcomes of the current review will be useful for mental health services and practitioners that aim to provide support for people with a sexual interest in children.

## Method

### Inclusion and Exclusion Criteria

Studies were included if they covered information about the treatment needs and/or experiences of pedohebephiles. This includes studies with information about (1) mental health needs; (2) incidence of participating in or wanting treatment for concerns related to sexual interest in children; (3) attitudes towards treatment; (4) experiences with treatment; (5) factors that facilitate or impede help-seeking; and (6) studies that report motives for dropout from clinical programs also qualified for inclusion. Studies were included if they presented information reported directly by pedohebephiles, as opposed to clinicians or third parties. In order to create a comprehensive overview of the field, we intentionally did not implement any filters for age, gender, years of publication, location, language, methods, context or type of assessment for pedohebephilia. In terms of population, nepiophiles, hebephiles, and pedophiles were included from community (i.e., participants drawn from the general population rather than specific clinical or forensic settings including online survey respondents), clinical (i.e., participants recruited from healthcare settings), and forensic settings (i.e., participants recruited from within the judicial or correctional systems) as well as mixed settings (i.e., studies that collect from multiple sources). Including forensic contexts alongside clinical or community settings may raise questions, as forensic samples often include individuals who deny sexual interest in children, despite their biography or psychophysiological responses suggesting otherwise. Despite this distinction, we decided to include forensic contexts to ensure that the review encompasses all relevant treatment contexts for pedohephiles, facilitating the comparison of treatment needs and experiences across a range of settings. Nevertheless, studies that classify people who have committed sexual offenses against children as “pedohebephiles” based solely on the young age of their victims (without further diagnostic procedures) were not included.

Studies were excluded if (1) they were case studies; (2) they were conducted on a different population (i.e., studies from the perspectives of clinicians, or studies that included ephebophiles in the same sample, the results of which could not be differentiated); (3) the method used was not an empirical study; (4) they did not have any information on treatment or needs; (5) attraction to children was not assessed in any way (i.e., self-report, DSM or ICD criteria, and indirect methods like viewing time or penile plethysmography).

### Literature Search

The initial database literature search was carried out in December 2022 using the databases ProQuest, PsycNET, PSYNDEX, PubMed and Web of Science, followed by manual searches of reference sections. The keywords utilized were: (pedophil* OR paedophil* OR nepiophil* OR hebephil* OR pedohebephil* OR minor-attract*) AND (“mental health” OR barrier* OR treat* OR support* OR psychotherap* OR “help-seek*” OR therap* OR prevent*).

A second database search was conducted in February 2024 at the request of the journal editor. The second search applied the same keywords and databases, only this time we filtered the search based on date of publication between December 2022 and March 2024. In addition to database searches, calls for grey literature were conducted periodically between December 2022 and August 2023. Grey literature was requested from prominent researchers in the field, by posting in the B4U-ACT Research Network forum as well as individually contacting authors from the B4U-ACT “Ongoing and Past Studies” page to inquire about potentially completed publications, and by posting a call for papers on SEXNET (sexnet@listserv.it.northwestern.edu).

### Characteristics of the Studies

#### First Literature Search

**Fig. 1:**
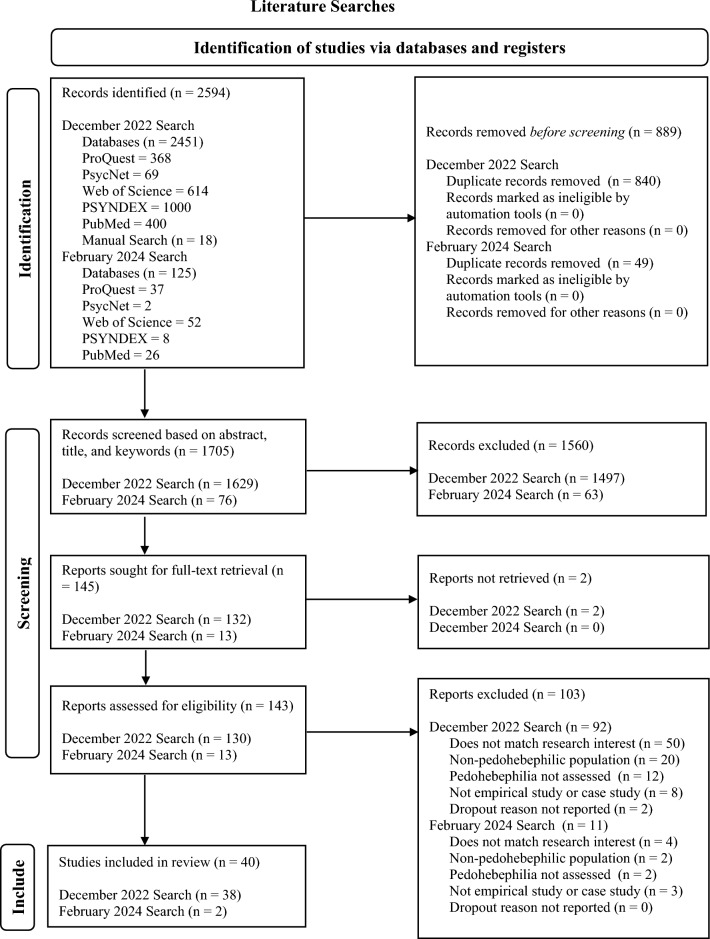
PRISMA flowchart

A total of 2,451 studies (see Fig. 1) were identified through the first database search, another 18 were found via manual searches, and duplicates were removed using EndNote Library. French and German publications were translated by one of the authors, while publications in other languages were done so using online document translation services. Records were uploaded to Covidence and 1629 titles and abstracts were screened. The remaining 129 reports underwent full-text screening and 92 publications were excluded, resulting in a total number of 37 studies included in this review, seven stemming from the manual search.

#### Second Literature Search

As a result of the second database search, an additional 125 studies were identified as having been published on the topic between December 2022 and March 2024. Using EndNote Library to cross reference with the first search, a total of 49 duplicates were removed. The remaining 76 reports were uploaded to Covidence for abstract and full-text screening, resulting in three studies included from the second literature search, bringing the total number to 40.

#### Inter-Rater Reliability

Two of the authors conducted a full screening of the literature using Covidence. Cohen’s κ for inter-rater reliability was calculated automatically by the software: for the title and abstract screening, there was a 94% agreement and a Cohen’s κ of 0.50 (moderate agreement) and for the full-text screening the reviewers had 85% agreement and a Cohen’s κ of 0.65, denoting substantial agreement. Disagreements were handled via discussion between the two authors until they reached consensus. There were no studies for which consensus could not be reached. Data from the final set of publications was extracted using a custom Covidence data extraction template (see Supplementary Materials). The data extracted included sample demographics, the research interest and pertinent information, the aim of the study, the design, methods and materials. Each author extracted data separately, and consensus was reached via the Covidence comparison interface.

#### Quality Assessment

A quality assessment was implemented on the final sample of studies. Initially, well-established frameworks such as that described in Caldwell et al. (2011) were considered. However, this was ultimately renounced in favour of a more tailored approach that better suits the particular challenges to study quality in this particular field. Thus, items were amended from another quality assessment developed for a meta-analysis on the difference between people with a sexual interest in children who have or have not committed sexual offenses (Chronos & Jahnke, 2023).

First, the lead author conducted an individual quality assessment of each paper. Next, the second author conducted an individual quality assessment of a random subsection of the studies comprising 50% of all articles, and sought consensus with the first author. Finally, the third author, having more experience with qualitative work than the other authors, conducted a check of the quality assessments for all qualitative and mixed methods studies and sought consensus with the first author. The full quality assessment (Table S1) can be found in the Supplementary Materials along with the scores (Table S2) for each study.

The results of the quality assessment were fairly positive overall, with the majority scoring over 70% and only a few papers scoring below 50%. Most studies fared well in terms of clear objectives and acceptable methods of participant recruitment, however, many studies struggled with acceptable representation of the target population as a result of recruiting self-referred participants. The quality of the quantitative and qualitative (or mixed) analyses varied greatly from study to study but the quantitative papers had lower scores overall, commonly due to many being of an exploratory nature, lacking explicit hypotheses and testing.

## Results

Most (70%) of the studies were conducted in a community setting with self-identified pedohebephiles, but several forensic, clinical, and mixed samples were also included (see Table 1 for a detailed overview of the study characteristics). Most were peer-reviewed, while seven were theses or dissertations (Extein, 2005; Freimond, 2013; Morris, 2023; Pedersen, 2023; Roche, 2020; Vogt, 2006; Walker, 2017) and one was a report (Stephens & McPhail, 2019). There were 22 qualitative, 15 quantitative, and three mixed methods studies. The most common method for assessing pedohebephilic interest was self-report (72.5%), followed by DSM-IV or DSM-5 criteria (25%) and structured assessments of risk and need treatment needs analysis frameworks (2.5%). There was only one population-based study (Dombert et al., 2016), the rest used ad-hoc samples. Four studies recruited from Dunkelfeld: Beier et al. (2009) collected self-report data from respondents between June 2005 and August 2008, Schaefer et al. (2010) from respondents between June 2005 and July 2007, Wagner et al. (2016) analyzed patient files between July 2005 and July 2013, and Stelzmann et al. (2022) analyzed data from Dunkelfeld patients between April and May 2018. There is, therefore, potential overlap between samples of the former three studies. However, the extent of the overlap is impossible to determine due to the methods used (surveys or interviews among former or current clients vs. patient files) and the anonymity standards applied. Due to this, we were unable to account for the overlap other than by marking the four studies mentioned as potentially susceptible to overlap in the results section and the tables.

**Table 1:** Study characteristics

Study	Source	*N*	Design	Sample	Offense Status	Exclusivity	Age	% Male
*Community*								
Beier et al., 2021	First search	3281	Quantitative	Troubled desire respondents	Both	–	Mode = 19–21	90.9
Bernard, 1975	First search	50	Quantitative	Dutch working group for pedophilia	Both	–	Range: 20–70	100
Cacciatori, 2017^G^	First search	7	Qualitative	Online self-identified pedohebephiles	No Offense	–	–	–
Dombert et al., 2016	First search	355	Quantitative	Population-based	Both	–	*M* = 42.18	100
Dymond & Duff, 2020	First search	3	Qualitative	Online self-identified pedohebephiles	No Offense	–	*M* = 34.6	100
Extein, 2005^G^	Manual search	6	Qualitative	Online self-identified pedohebephiles	Unknown	–	Range: 29–62	100
Freimond, 2013^G^	Manual search	9	Qualitative	Online self-identified pedohebephiles	Unknown	–	Range: 20–70	100
Houtepen et al., 2016	First search	15	Qualitative	Online self-identified pedohebephiles	Both	40%	–	100
Ingram et al., 2024	Second search	15	Qualitative	Online self-identified pedohebephiles	No Offense	26%	*M* = 36	73
Jahnke et al., 2015	First search	104	Quantitative	Online self-identified pedohebephiles	Both	68%	*M* = 37.30	100
Jahnke et al., 2023	Manual search	136	Mixed	Online self-identified pedohebephiles	Unknown	85.3%	*M* = 34.35	80
Jimenez-Arista & Reid, 2023	First search	61	Qualitative	Analysis of forum posts	No Offense	–	–	–
Lievesley et al., 2020	First search	183	Quantitative	Online self-identified pedohebephiles	Unknown	73%	*M* = 33.17	90
Lievesley et al., 2023	First search	150	Quantitative	Online self-identified pedohebephiles	Unknown	80.7%	*M* = 32.8	91
Mitchell & Galupo, 2018	First search	100	Mixed	Online self-identified pedohebephiles	Both	–	Range: 18–65	95
Morris, 2023	Second search	17	Qualitative	Online self-identified pedohebephiles	Unknown	–	Range: 19–75 +	70.6
Moss et al., 2021	First search	293	Quantitative	Online self-identified pedohebephiles	Unknown	–	*M* = 31.61	89
Pedersen, 2023^G^	Manual search	5	Qualitative	Online self-identified pedohebephiles	Unknown	–	–	100
Roche, 2020^G^	Manual search	183	Qualitative	Online self-identified pedohebephiles	Unknown	–	*M* = 32	84.4
Roche et al., 2022	First search	353	Quantitative	Online self-identified pedohebephiles	Unknown	–	*M* = 35	89.4
Schaefer et al., 2023	First search	319	Qualitative	Online self-identified pedohebephiles	Both	34.1%	*M* = 35.44	94
Shields et al., 2020	First search	30	Qualitative	Online self-identified pedohebephiles	Unknown	–	Range: 18–30	92.9
Stephens & McPhail, 2019^G^	Manual search	290	Quantitative	Online self-identified pedohebephiles	Unknown	42.3%	*M* = 31.7	89.6
Stevens & Wood, 2019	First search	5210	Qualitative	Analysis of forum posts	Unknown	–	–	–
Tozdan et al., 2022	First search	52	Quantitative	Online self-identified pedohebephiles	Both	10%	*M* = 33.2	7.7
Tozdan et al., 2023	Manual search	50	Qualitative	Online self-identified pedohebephiles	Unknown	–	*M* = 33.6	0
Walker, 2017^G^	First search	41	Qualitative	Online self-identified pedohebephiles	No Offense	34%	Range: 20–50	88
Wilpert & Janssen, 2020	First search	312	Quantitative	Analysis of contact logs	Both	7.6%	*M* = 36.90	94.5
*Clinical*								
Beier et al., 2009	First search	314	Quantitative	Dunkelfeld respondents	Both	55%	*M* = 38.8	100
Landgren et al., 2020	First search	52	Quantitative	PrevenTell respondents	Both	21%	Range: 18–66	100
Schaefer et al., 2010	First search	160	Quantitative	Dunkelfeld respondents	Both	–	*M* = 35.52	100
Stelzmann et al., 2022	First search	20	Qualitative	Dunkelfeld respondents	Unknown	–	–	–
Wagner et al., 2016	First search	186	Qualitative	Dunkelfeld respondents	Both	–	-	100
*Forensic*								
Blagden et al., 2018	First search	20	Qualitative	Incarcerated pedohebephilic child sex offenders	Offense	100%	Range: 18–36	100
Drapeau 2005a	First search	15	Qualitative	Psychiatric inpatient pedohebephilic offenders	Offense	–	*M* = 44.13	100
Drapeau et al., 2005b	First search	23	Qualitative	Psychiatric inpatient pedohebephilic offenders	Offense	–	*M* = 44	100
Walton & Duff, 2017	First search	5	Qualitative	Incarcerated pedohebephilic child sex offenders	Offense	–	Range: 24–50	100
*Mixed*								
Boons et al., 2021	First search	12	Qualitative	Psychiatric outpatient pedohebephilic offenders	Offense	–	Range: 25–74	100
Tozdan & Briken, 2019	First search	120	Quantitative	Post-hoc analysis of outpatient and online data	Both	19%	*M* = 38.15	100
Vogt, 2006^G^	First search	72	Mixed	Purposive sampling of pedohebephiles	Both		*M* = 38.5	100

^G^–Grey literature

The offense status of the participants was often either mixed (37.5%) or unknown (37.5%). Five (12.5%) studies were conducted exclusively on pedohebephiles who have not perpetrated sexual offenses and five (12.5%) exclusively on pedohebephiles who have perpetrated sexual offenses. The majority of samples were comprised almost entirely of participants of male gender identity, with few exceptions (Ingram et al., 2024; Jahnke, et al., 2023; Morris, 2023; Tozdan et al., 2022, 2023). Eighteen (45%) of the studies were conducted via interviews, 18 (45%) were surveys, three (7.5%) were document analyses, and one (2.5%) was a focus group discussion. Three (8%) of the studies were published in German (Beier et al., 2021; Vogt, 2006; Wagner et al., 2016) and the rest were published in English. Based on the data extracted, we categorized the findings into four overarching categories: Treatment Interest, Treatment Motives, Treatment Experience, and Barriers and Facilitators to Treatment (see Table 2).

**Table 2:** Studies and included categories

Category				
Publication	Treatment interest	Treatment motives	Treatment experience	Treatment barriers and facilitators
*Community*				
Beier et al., 2021		X		
Bernard, 1975	X	X		
Cacciatori, 2017	X	X	X	X
Dombert et al., 2016	X			
Dymond & Duff, 2020			X	X
Extein, 2005			X	
Freimond, 2013			X	X
Houtepen et al., 2016	X	X	X	X
Ingram et al., 2024			X	X
Jahnke et al., 2015	X			X
Jahnke et al., 2023		X	X	X
Jimenez-Arista & Reid, 2022	X		X	X
Lievesley et al., 2020	X			
Lievesley et al., 2023		X		
Mitchell & Galupo, 2018			X	X
Morris, 2023			X	X
Moss et al., 2021		X		
Pedersen, 2023				X
Roche, 2020	X	X	X	X
Roche et al., 2022				X
Schaefer et al., 2023	X	X		X
Shields et al., 2020				X
Stephens & McPhail, 2019	X	X	X	**X**
Stevens & Wood, 2019	X	X	X	
Tozdan et al., 2022	X	X		
Tozdan et al., 2023			X	X
Walker, 2017	X	X	X	X
Wilpert & Jansen, 2020	X			
*Clinical*				
Beier et al., 2009	X			
Landgren et al., 2020			X	
Schaefer et al., 2010	X	X		
Stelzmann et al., 2022				X
Wagner et al., 2016			X	
*Forensic*				
Blagden et al., 2018			X	X
Drapeau 2005a		X		X
Drapeau et al., 2005b		X	X	X
Walton & Duff, 2017				X
*Mixed*				
Boons et al., 2021			X	
Tozdan & Briken, 2019	X			
Vogt, 2006	X	X	X	

### Treatment Interest

Seventeen studies assessed the interest or participation in treatment (see Table 3). Of the 12 that reported on interest, the majority (*n* = 7) demonstrated moderate (35–70%) to high (over 70%) incidence while only three reported relatively low (under 35%) levels of interest. The highest scores estimate is unreliable due to the small sample size (Cacciatori, 2017; *n* = 7). A desire to change their sexuality was considered as interest in treatment due to its perception by participants as being a potential goal of treatment. Tozdan and Briken (2019) found that their entire outpatient subsample (*n* = 26) wanted to change their interest to some degree, and 50% reported that they want to completely. Only 11.8% of the forensic and 9.1% of the non-forensic sample felt they did not want to change their sexual interest at all, and 47.1% of forensics and 38.6% of non-forensics reported complete agreement. There was an inverse trend in the internet subsample, however, where the majority reported that it did not apply to them (48.5%).

**Table 3:** Interest and participation in treatment

Study	% Interested in Treatment	% Participated in Treatment
*Community*		
Bernard, 1975	–	38%
Cacciatori, 2017^a^	85%	57.1%
Dombert et al., 2016	13.9%	–
Houtepen et al., 2016	–	86%
Jahnke et al., 2015	52%	–
Jimenez-Arista & Reed, 2023^b^	Some users	–
Lievesley et al., 2020^c^	Need support: 2.17	–
Roche, 2020	68.3%	52.8%
Schaefer et al., 2023	8.4%	–
Stephens & McPhail, 2019^d^	–	26.2%
Stevens & Wood, 2019	–	27%
Tozdan et al., 2022	42.3%	29%
Walker, 2017^e^	85%	
Wilpert & Jansen, 2020	42%	14.4%
*Clinical*		
Beier et al., 2009^f^	26%	46.5%
Schaefer et al., 2010	–	45%
*Mixed*		
Tozdan & Briken, 2019	81.6%	53.2%
Vogt, 2006	–	52.8%

^a^Cannot be a reliable estimate based on *n* = 7 (Cacciatori, 2017).

^b^Jimenez-Arista and Reid (2023) did not report a statistic.

^c^Lievesley et al. (2020) asked participants whether they felt they needed more support and the mean score was 2.17 on a scale of 1 (do not need support) to 3 (need support).

^d^A large proportion (39.8%) of participants surveyed by Stephens and McPhail (2019) reported that they did not seek mental health treatment because they did not feel distressed or that they required it.

^e^Walker (2017) reported a combined statistic.

^f^Beier et al. (2009) reported data from subsamples. 26% of a subsample of *n* = 146 were interested in treatment and 46.5% of a subsample of *n* = 273 had consulted their GP or a practitioner in the last 6 months for their sexual interest.

Thirteen studies reported on participation in treatment (either currently, or in the past); the majority (*n* = 9) found moderate to high incidence while only three found incidences under 35%. The highest participation rates were found in the community samples, followed by clinical and mixed samples, where approximately half of participants had previously been in treatment. Part of Vogt’s (2006) sample was forensic and had to attend compulsory treatment, and Tozdan & Briken’s sample (2019) was a mix of post-hoc data from clinical, community, and forensic participants, thus it is unclear to what degree their participation was voluntary. The smallest proportion (26.2%) of pedohebephiles having attended therapy was found in Stephens and McPhail’s (2019) study where a large proportion (39.8%) of participants reported that they did not seek mental health treatment because they did not feel distressed or that they required it. This was followed by 27% of pedohebephiles having attended therapy in Stevens and Wood (2019), although this could be due to the lack of spontaneous disclosure on the topic found in online forum posts. In the studies that reported on both participation and interest in treatment, there were nearly always more participants that were interested than had participated. In Roche (2020), 52.8% of the sample had participated in treatment compared to the 68.3% who were interested in it. The trend followed for Tozdan et al., (2022; 29% participated vs. 42.3% interested), Wilpert and Janssen (2020; 14.4% participated vs. 42% interested), and Tozdan and Briken (2019; 53.2% participated vs. 81.6% interested). This suggests that there are perhaps barriers impeding this community from seeking help.

### Treatment Motives

Seventeen studies reported on treatment motivations related to participants’ sexual interest in children (see Table 4). Pedohebephiles most commonly reported needing treatment or support for mental health problems, distress related to their sexual interest, depression, anxiety, suicidality, addiction, to get help coping with their sexual attraction, and/or addressing the effects of social stigma. There were marked differences, however, between sample types. While participants from a community setting were more likely to report needs related to their mental health, participants from clinical and forensic setting reported seeking treatment due to pressures felt from their families, friends, or partners (Drapeau et al., 2005b). They had multiple motives for entering treatment related to legal repercussions such as mandated treatment, entering treatment because of an ongoing or recent criminal case, or the inability to abstain from using CSAM (Drapeau et al., 2005b). Finally, forensic and clinical (or mixed) samples more commonly reported needing support with abstaining from offending (Schaefer et al., 2023), or gaining a sense of mastery over their actions (Drapeau et al., 2005b), than did community samples.

**Table 4:** Treatment motives

Study	Motives
*Community*	
Beier et al., 2021	Distress, problems related to sexual interests
Bernard, 1975	Changing attraction
Cacciatori, 2017	Distress, depression and hopelessness, anxiety, suicidality
Houtepen et al., 2016	Accepting and/or coping with attraction, depression, loneliness
Jahnke et al., 2023	Desperation, emotional turmoil, depression, coping, validation
Lievesley et al., 2023	Mental health, stigma, sexual frustration, controlling or changing attraction
Moss et al., 2021	Coping, stigma
Roche, 2020	Mental health, coping, stigma, abstaining from offending, managing romantic relationships, disclosing attraction
Schaefer et al., 2023	Mental health, more information on attraction
Stephens & McPhail, 2019	Problems related to sexual interests
Stevens & Wood, 2019	Managing mood, addiction, anxiety, depression, self-hatred, self-harm, suicidality
Tozdan et al., 2022	Changing attraction
Walker, 2017	Mental health, depression, anxiety, suicidality, resilience against offending, changing attraction
*Clinical*	
Schaefer et al., 2010	Mental health, distress, perceived risk of reoffending
*Forensic*	
Drapeau 2005a	Recovering freedom, gaining mastery, avoiding criticism and rejection, acceptance
Drapeau et al., 2005b	Coping, acknowledging reality, pressure from family, pressure from treating staff
*Mixed*	
Vogt, 2006	Improvement of capabilities, finding meaning and contentment, dealing with affective disorders, coping with sexuality

There was one motive for seeking treatment that was highly contentious, namely, changing their sexual attraction to children. Multiple studies (see Table 4) reported that participants had sought or wanted to seek help with the goal of changing their attraction to children. However, as time went on, many came to realize that their attractions were enduring and redirected their goals towards managing them and finding ways to live productive and meaningful lives (Walker, 2017). Still, the notion that pedohebephilia can be cured or altered in some way is a recurring theme for many people with a sexual interest in children, and comes up in the following sections when discussing their experiences with treatment as well as barriers and facilitators. In addition to self-reported motives, some studies investigated factors that were correlated with motivation and found that maladaptive coping, internalized sexual stigma (Moss et al., 2021), and low psychological well-being were associated with a greater desire for treatment and support (Lievesley et al., 2020).

### Treatment Experiences

Twenty studies reported on the experiences of pedohebephiles in treatment (see Table 5). There were no substantial differences between samples in the incidence of positive or negative experiences; however, there were differences in the nature of their experiences. Specifically, some of Vogt’s (2006) sample reported that therapy was compulsory and thus perceived negatively. Drapeau et al. (2005a) participants reported negative experiences in the context of group therapy. They also felt their risk of recidivism was not declining in spite of the therapeutic process. Some of Morris’ (2023) sample and one participant from Wagner et al. (2016) reported that they had previously (not during the studies in question, but in their past help-seeking experiences) received some form of aversion therapy which they found to be very negative. Aversion therapy is a psychological treatment designed to reduce or eliminate sexual arousal to children by associating it with negative stimuli or experiences (McPhail & Olver, 2020).

**Table 5:** Treatment experiences

Study	Positive	Negative
*Community*		
Cacciatori, 2017	Therapist who listens and avoids making assumptions	Lack of effective and available help, feelings of hostility, unsafety, abandonment
Dymond & Duff, 2020	–	Services based on repressing instead of accepting sexuality
Freimond, 2013	Comfort in talking with counsellors about attraction about human sexuality and ranges of sexual attraction	–
Houtepen et al., 2016	Therapy helped them accept attraction and deal with stigma	Perceived lack of professional knowledge
Ingram et al., 2024	–	Therapist focus on prevention, rejection, feelings of hostility
Jahnke et al., 2023	–	Reported to police and social services, rejection
Jimenez-Arista & Reid, 2023	–	Inexperienced therapists, rejection
Mitchell & Galupo, 2018	–	Abandonment by counselor after confessing attraction
Morris, 2023	Found support in processing what it means to be attracted to children. Found therapy specific to pedohebephiles to be helpful and affirming	Aversion therapies. Rejection, breach of confidentiality, invalidation, shaming
Roche, 2020	53.5% found counselling experience to be positive	46.5% found counselling experience to be negative
Stephens & McPhail, 2019	26.2% of the sample that sought help for their sexual interest in children. The average rating of their experience was a 5.6 out of 10	
Stevens & Wood, 2019	Abstinence from offending, reduced attraction, improved mental health, less suicidality	–
Tozdan et al., 2023	Non-judgmental therapist, helpful, genuine interest and empathy, not being alone	Therapy was not helpful, experienced disbelief, rejection, disgust, distrust of professional
Walker, 2017	Effective, nonjudgmental mental health care	Misunderstandings, suspicion, loss of privacy, inexperienced therapists, “curing” pedohebephilia, treating them as “walking time bombs”
*Clinical*		
Landgren et al., 2020	Positive effects on sexuality, relationship, mental health, changed perspective, improved cognitive ability, self-control, improved physical health	Negative effects on body and sexuality, relationship problems, mental health issues, decreased cognitive ability, negative emotions and effects on work
Wagner et al., 2016	–	Rejection, inexperience, guilt, repression, aversion therapy
*Forensic*		
Blagden et al., 2018	Methods of tackling attraction head-on	–
Drapeau et al., 2005a, 2005b	–	Too many participants in group therapy, therapist disbelief, risk of recidivism despite completion of therapy
*Mixed*		
Boons et al., 2021	Abstinence from offending, calming effect, positive influence on general well-being	Sexual dysfunction, physical side effects, depression, guilt
Vogt, 2006	30% found psychotherapy to be helpful or very helpful	A third of participants reported that it was not helpful, or that it had been “forced treatment.”

Participants from studies classified as clinical, forensic, or mixed, reported on experiences with chemical treatments such as androgen deprivation therapy (Boons et al., 2021) or gonadotropin releasing hormone antagonists (Landgren et al., 2020). In these cases, positive experiences were largely reported to be the calming effects of the chemicals, abstinence from offending, and improved mental health and well-being. The negative experiences included physical side effects, depression, and guilt. Interestingly, within these samples, the inability to become aroused was reported as a positive treatment effect by some and a negative treatment effect by others. In the studies on community samples, positive experiences were reported when participants had access to non-judgmental care with a therapist they felt listened to them. According to participants, these experiences led to improved mental health, cognitive ability, and self-control, among others. Common negative experiences included feelings of rejection and hostility, the therapists’ perceived lack of competence with pedohebephilia, and treatment goals that do not align with the goals of the client (i.e., the client is interested in learning coping skills, yet the therapist is prevention-oriented) (Dymond & Duff, 2020; Ingram et al., 2024).

### Treatment Barriers and Facilitators

Barriers and facilitators to treatment was the most commonly researched category in this review (see Table 6), and the results were overwhelmingly skewed towards barriers. The factors that pedohebephiles discussed as facilitators of treatment included knowing a therapist had experience with pedohebephilia and provided a safe and empathetic environment. In addition, some participants mentioned more general factors outside the therapeutic environment that would have encouraged them to seek help, and these included dispelling negative messaging in media and support campaigns aimed at pedohebephiles and replacing them with hopeful messaging (Jahnke et al., 2015). Others reported that reading testimonials from previous patients or clients of support and prevention organizations was a potential motivator for seeking help themselves.

Barriers were commonly marked by fears relating to the repercussions of seeking treatment. Many participants were afraid that they would be reported or outed, and thus lose their livelihoods, autonomy, and relationships. Others feared rejection and stigmatization from the theapist, with some participants in Jahnke et al. (2023) feeling stigmatized as a result of receiving prevention-aimed offers. Other barriers included lack of information about resources or financial and geographical inaccessibility. Finally, pedohebephiles reported that they felt there was a lack of professional resources available, and called into question the quality of said resources in treating pedohebephilic individuals specifically. There were limited differences between sample types regarding barriers and facilitators, with the brunt of them focused on the specifics of group therapy in forensic contexts and a perceived lack of continuity in support when leaving the prison system.

**Table 6:** Treatment barriers and Facilitators

Study	Barriers	Facilitators
*Community*		
Cacciatori, 2017	Feeling unsafe, fear of exposure, rejection, being misunderstood, labelling, status loss, disconnection from society, stigma	Mental health practitioners that are open, welcoming, empathetic, listen and adapt their treatment goals accordingly
Dymond & Duff, 2020	Inaccessibility, distrust, fear of being reported	–
Freimond, 2013	Perceived hysteria from professionals, fear of being reported	–
Houtepen et al., 2016	Perceived lack of clinical knowledge about pedohebephilia, stigmatization	–
Ingram et al., 2024	Distrust, reluctance to disclose attraction	–
Jahnke et al., 2015	Perceived lack of understanding, expect negative reaction	–
Jahnke et al., 2023	Prevention focus, distrust, fear of being reported, fear of rejection	Compassionate therapist, experienced with pedohebephiles
Jimenez-Arista & Reid, 2023	Financial barriers, fear of being reported, fear of rejection	–
Mitchell & Galupo, 2018	Abandonment, lack of clinical knowledge about pedohebephilia	–
Morris, 2023	Anxiety, fear of exposure, fear of what they might learn	–
Pedersen, 2023	Fear of exposure, lack of information, stereotyping, stigma	–
Roche, 2020	–	Differentiating interest and behavior and pedophilia vs. pedophilic disorder, awareness of sexological research, transparent reporting protocol, promoting positive identity and pro-social relationships, addressing stigma, confidentiality
Roche et al., 2022	Fear of seeking help, lack of professional help	–
Schaefer et al., 2023	Fear of being reported, arrested, ostracized, lack of known resources, distrust and alienation	–
Shields et al., 2020	Negative messaging—inevitability of offending	Dispelling negative messages and promoting positive messages
Stephens & McPhail, 2019	Fear of stigmatization, fear of being reported, inability to share problems with others, negative attitude towards treatment providers, desire to handle problem themselves, financial barriers, inaccessibility	–
Tozdan et al., 2023	–	Acceptance, destigmatization, awareness of the existence of female pedohebephiles, media/advertising, treatment features, assurance of anonymity, special treatment programs for females, supportive therapists, no enforcement of sex with adults, legalization of sexual contacts with children
Walker, 2017	Fear of being reported, fear of being outed, loss of safety and privacy, fear of judgment and stigmatization. Financial barriers	Experience with pedohebephilia, not “future offenders.”
*Clinical*		
Stelzmann et al., 2022	Media reports increase stigma and prevent help-seeking	Reports from prevention organizations featuring testimonials from patients and content of the program
*Forensic*		
Blagden et al., 2018	Lack of information	–
Drapeau et al., 2005a	Authoritarian therapists, disrespectfulness, rejection, criticism	Safe and predictable environments
Drapeau et al., 2005b	Insufficient individual attention, critcism, devaluation, fear of being hurt by group, shame and rejection	Organized and program structure, security, ability to talk freely, feeling respected. Therapists that show authority, leadership, and strength, willing to listen, answer questions, provide explanations
Walton & Duff, 2017	Lack of professional support outside prison, no genuine help offered, labelling	–

## Discussion

To our knowledge, this is the first systematic review to investigate the self-reported treatment needs and experiences of people with a sexual interest in children. The desire for support was evident (Houtepen et al., 2016; Schaefer et al., 2023; Tozdan & Briken, 2019; Walker, 2017) and the diverse motives for seeking treatment reflected the complex challenges faced by pedohebephiles. Some reported positive treatment outcomes, such as improved mental health, cognitive abilities, and self-control (Landgren et al., 2020). However, negative experiences such as rejection and hostility were common (Drapeau et al., 2005b; Jimenez-Arista & Reid, 2023). Fear of being reported or outed and concerns about legal repercussions were significant barriers for many (Jimenez-Arista & Reid, 2023), while therapists with experience with pedohebephiles, safe and empathetic environments were considered to encourage help-seeking (Cacciatori, 2017).

Most of the studies were conducted on community samples of online self-identified pedohebephiles via forums such as B4U-ACT and VirPed. Clinical and forensic samples were less common, and there was only one population-based sample. Despite the different samples, findings converged with respect to participation in treatment which was not uncommon in community, clinical, and mixed samples. Common motives for treatment included coping with distress and finding acceptance, while common experiences in treatment were rejection from the therapist and a misalignment of the client’s and therapist’s goals for treatment. Some barriers (perceived lack of information or availability of support resources) and facilitators (having a safe and predictable environment) were also shared between different samples.

There were, however, marked differences in the needs and experiences of people with a sexual interest in children from different samples. Clinical and forensic samples tended to be motivated to seek treatment by incarceration-related factors (i.e., to regain their freedom in court-mandated cases, to gain mastery over their impulses and abstain from reoffending). They also reported feeling pressure from family and even staff to enter treatment. This was never the case with community samples, the motives of which were more centered on their mental well-being and gaining the skills to live fulfilling lives. The experiences in treatment of those in clinical and forensic samples were often focused on a specific treatment program (the pros and cons of group therapy, the effects of chemical treatments). Community samples, having typically had no experience in such contexts, more often discussed the common factors in treatment such as empathy, openness, and specialized knowledge. Finally, in terms of barriers and facilitators, clinical and forensic samples once again reported factors specific to their contexts such as the pros and cons of the treatment programs they were participating in and the lack of continuity in treatment after incarceration. Community samples discussed common factors in treatment, or lack thereof.

The discrepancy between different samples was due to the limited number of clinical and forensic samples that included the perspectives of pedohebephiles, as opposed to, e.g., outcome measures assessing mental health or risk factors for child sexual offending. This may in part be due to participants in forensic settings denying pedohebephilic interest. In this case, it would not make sense to ask study participants about barriers to seeking treatment for a sexual interest that they deny having. It may also indicate pervasive social stigma related to pedohebephilia and sexual offending, which may contribute to distrust towards this population, reluctance to assess their perspectives, or lack of advocacy or public funding for such research efforts. However, it is less clear why few studies in forensic settings assessed study participants’ perception of or experiences with the treatment that was provided. The absence of such research may inhibit the development of more tailored interventions, which may increase the effectiveness of psychotherapy programs.

The results of the current review also stand in contrast to the perspectives of practitioners in regard to treatment. For instance, Bayram et al. (2023) found that health care practitioners’ main goal would be preventing child sexual abuse, followed by understanding pedophilia, increasing quality of life, protecting society, and ceasing the use of CSAM. When asked about the goals of their pedohebephilic patients, they reported that preventing harm would be the first on the list, followed by changing sexual interest, understanding pedophilia, using treatment as an excuse to justify immorality, and finding companionship. Similarly, Lievesley et al. (2023) found that practitioners valued controlling behavior much more highly than pedohebephiles did. When it comes to barriers, there seems to be a consensus between practitioners and the perspectives of pedohebephiles. Fear of disclosure due to personal and legal consequences, as well as lack of availability of professional help (or knowledge of where it can be found) are commonly reported as the main concerns when seeking treatment by therapists (Parr & Pearson, 2019). The practitioners in the study also go on to suggest that these barriers may be reduced by increasing publicity, education and training regarding pedohebephilia. These improvements align with some of the facilitators synthesized in the present literature review, such as having knowledgeable and empathic clinicians. Additionally, Goodier and Lievesley, (2018) looked at the needs of individuals at risk from practitioners’ perspectives and reported lack of trust in services as the main barrier to intervention, followed by anonymity – that many individuals at risk are undetected and can therefore not be reached for intervention. Although here seems to be a mutual understanding regarding the barriers to seeking help, one commonly identified barrier is, in fact, the discrepancy between the treatment goals of the patient and the practitioner, fueled at least in part by a misunderstanding of the motives for which people with a sexual interest in children want to seek help in the first place. Recognizing that the motives for which pedohebephile seek treatment are as diverse as any other individual is a first step in bridging the gap and offering effective support.

### Strengths and Limitations

While the review offers valuable insights, it is essential for readers to understand its inherent limitations. Publication bias occurs when the decision to publish a study is influenced by the direction or significance of the study’s findings and is a frequent problem in literature reviews (Borenstein et al., 2021). Although we have done our best to circumvent publication bias via manual searches and the inclusion of grey and non-English literature, it is still possible that relevant literature could have eluded our efforts to identify it. The second limitation is that the majority of participants in the studies were self-identified pedohebephiles recruited online via forums and networks such as B4U-ACT and VirPed, thus resulting in a potentially significant degree of overlap between the samples. In addition, these samples are more likely to capture participants with a specific profile, (i.e., non-offending, seeking support, etc.). Results may differ if based on other forums or pedohebephiles who do not engage is such forums at all. The only population-based sample (Dombert et al., 2016) found the lowest rate of interest in treatment, which could indicate that the studies based on community members may inflate that figure. Third, the quality assessment of the included studies revealed methodological challenges such as poor representation of the target population due to recruitment through self-referral, and lack of rigor in data collection and analysis in both quantitative and qualitative reports (see Table S1 in Supplementary Material).

It could be argued that our criteria of excluding ephebophilic individuals may be a limitation. Indeed, some researchers in this field prefer an inclusion of a wider set of sexual interests, as subsumed under the term “minor attraction” (Grady et al., 2019; Levenson & Grady, 2019). Others, like us, prefer to focus on a more narrow set of interests, typically including pedophiles and hebephiles (Jahnke et al., 2022b; McPhail & Olver, 2020; Seto, 2018), as sexual attraction to postpubescent partners that are (or appear) youthful is neither rare nor unusual in the general male population (Miller & McBain, 2022). Even so, studying the treatment needs and experiences of people who report attraction to postpubescent minors could be insightful for future reviews, as there are indications that they experience similar barriers to treatment than pedohebephiles (Grady et al., 2019; Levenson & Grady, 2019). Furthermore, the majority of studies have been conducted with Western samples, most of which were English-speaking online communities. This suggests a potential lack of generalizability to non-Western samples or those less likely to be found on online forums. Strengths of the current review include the large number of included studies and the minimization of selection bias via the broad systematic search (including research published in non-English languages and grey literature), required reviewer consensus from screening to extraction, and substantial inter-rater reliability.

### Implications for Research and Clinical Practice

With respect to community-based treatment, some experts have proposed to balance well-being goals and prevention goals, but the extent to which either should take precedence remains contested. The finding that, at least in community settings, few participants appear to have an interest in prevention goals, such as learning how to control or reduce their sexual attraction to children, therefore poses practical and ethical challenges for treatment providers. One way to balance the goals of offense prevention and individual well-being could be the use of the Good-lives-model, which seeks to encourage individuals to pursue meaningful and prosocial life goals (Willis & Ward, 2013), rather than deficit-oriented approaches like relapse prevention. However, it stands to reason that there should be more services with a stronger or even exclusive commitment to well-being goals, given that there are pedohebephiles with low risks of sexual offending.

Furthermore, this review has identified that fear of rejection, fear of being reported and lack of trust are significant barriers to help-seeking for people with a sexual interest in children. These are important considerations for any therapist working with this client group, one which may make disclosures about sexual interests that would typically expose them to stigma and moral outrage (Scrivens & Ricciardelli, 2019). As highlighted in the review it is important for pedohebephiles to have a safe space in which to share experiences, particularly as it is likely to evoke significant levels of stigma, shame, and judgment (Wagner et al., 2016). The onus, then, is on the therapist to create a safe and non-judgmental environment which fosters a therapeutic relationship characterised by warmth, respect, genuineness, and empathy (Patterson, 1984). The therapist contribution to the therapeutic alliance, how supportive it is perceived and how trusting it is experienced, is crucially important for psychotherapeutic outcomes (Del Re et al., 2012). Patterson (1984) concluded that evidence for the necessity of therapist displays of empathy, respect and warmth was “incontrovertible” (p. 437). Thus, therapists working with this client group need to understand their mental health and treatment needs in order to provide effective, ethical, and compassionate services for this stigmatised and hard-to-reach population.

Creating a compassionate and non-shaming therapeutic environment is especially important to pedohebephiles in order for them to share openly their experiences, and the impact their sexual attraction has had on them (Hocken &Taylor, 2021). One form of therapy which appears particularly well suited to this client group is Compassion Focused Therapy (CFT) (Hocken & Taylor, 2021). CFT was initially developed for people whose elevated levels of shame rendered them unable to benefit from traditional CBT (Gilbert, 2014). CFT can be understood as a motivation focused therapy, based on evolutionary and cognitive systems, which helps people to access and stimulate the affiliative emotions, motives and competencies underpinning compassion. The combination of these capacities plays a significant role in threat regulation, well-being, and pro-social behavior (Gilbert, 2014; Hocken & Taylor, 2021). Within CFT, the relationships individuals have with themselves, especially in the forms of shame and self-criticism—highly relevant to pedohebephiles—underpin a wide range of mental health problems (Gilbert, 2014). There is emergent evidence that compassion-based interventions can reduce shame and help pedohebephilic individuals towards meaningful clinical change (Clayton et al., 2022). As Gilbert (2014) contends “compassion moves us to wanting to take responsibility for change and do what we can to engage with and help with the suffering of ourselves and others” (p. 30).

### Future Directions

As the first systematic review on this topic, the present research was conducted with the aim of providing a comprehensive overview on pedohebephiles’ treatment needs and experiences with no filters for publication year, context (community vs. clinical vs. forensic), participant sex, or other aspects or conditions which could affect participants’ treatment needs or experiences. Nevertheless, we hope that this broad overview will serve as a foundation for future research to identify specific areas for more targeted literature reviews, possibly including meta-analysis.

Future research should aim to report the perspectives of patients regarding treatments as opposed to only outcomes such as recidivism or offending behavior. In this way, the success of future interventions can be measured as a comparison between the fulfillment of clients’ goals and that of the practitioners. Further investigation is also warranted into the needs and experiences of pedohebephiles stemming from different settings and how treatment goals and strategies can be adapted to this end. Strikingly, the present review was only able to identify a few studies from a forensic context that have assessed participants experiences with or attitudes towards treatment, and among the few, the extent to which their perspectives were included was minimal. This is unfortunate, as client experiences could give important clues as to how the effectiveness of treatment could be improved. Finally, it would be highly beneficial for any future study in the field to report outcomes separately based on attraction to different age groups (as well as exclusivity of attraction) of their participants to investigate any potential similarities and differences.

### Conclusion

Although the literature on treatment of people who are sexually attracted to children has grown considerably in the recent decade (Landgren et al., 2022), there is still much uncertainty around what constitutes best practice for this group, particularly in non-mandated settings. By understanding the perspectives and experiences of people who are sexually attracted to children, mental health services can be better equipped to provide appropriate and effective support, ultimately contributing to the well-being of both pedohebephilic individuals struggling with these attractions as well as the broader community.

## Supplementary Information

Below is the link to the electronic supplementary material.

Supplementary file1 (DOCX 32 KB)

## Data Availability

The screening data is not publicly available.
