# Supplementary material for: The Treatment Needs and Experiences of Pedohebephiles: A Systematic Review
Source: Arch Sex Behav. 2024 Jul 15;53(9):3329–46. doi: 10.1007/s10508-024-02943-0 (PMC11390808; doi:10.1007/s10508-024-02943-0)
Supplement: Supplementary file 1 — Supplementary file1 (DOCX 32 KB) [file 10508_2024_2943_MOESM1_ESM.docx]

Supplementary Materials

Table S1. Quality Assessment

| **Assessment** | **Guidance** | **Rating** |
| --- | --- | --- |
| Q1. Were the objectives clear? | Yes = The objectives were clear.  Partially = The objectives were somewhat clear.  No or N/A = The objectives were not clear and/or the purpose of the study was something other than to assess the treatment needs and experiences of pedohebephiles. | Yes = 2  Partially = 1  No or N/A = 0 |
|  |  |  |
| Q2. Were the participants recruited in an acceptable way? | Yes = Participants were appropriately selected, recruitment process described, and ethical principles adhered to.  Partially = Recruitment process is described but important details are missing.  No = No recruitment process described. | Yes = 2  Partially = 1  No = 0 |
|  |  |  |
| Q3. Are the individuals selected to participate in the study likely to be representative of the target population? | Yes = Participants from population-based sample or systematically selected from a source list (i.e., support organization, clinic, prison, mental health facility).  Partially = Self-referred from multiple sources.  No = Self-referred from only one source or participant characteristics are not appropriately described. | Yes = 2  Partially = 1  No = 0 |
|  |  |  |
| Q4. Was the quantitative data collected in a way that is valid and reliable? | Yes = The methods of data collection are valid and reliable or not applicable (e.g., general sociodemographic factors, treatment attendance).  Partially = There are some indicators for validity or reliability or some measures are valid and reliable.  No = The measures’ validity or reliability is lacking, unclear or (for new or not well-established measures) not described. | Yes = 2  Partially = 1  No = 0 |
|  |  |  |
| Q5. Was the method of data analysis sufficiently rigorous? | Yes = The method of data analysis used was appropriate for the research design and question.  Partially = Some statistical tests used were appropriate for the research design and question.  No = Statistical tests used were not appropriate for the research design and question. | Yes = 2  Partially = 1  No = 0 |
|  |  |  |
| Q6. Was the qualitative data collected in a way that addressed the research issue? | Yes = The method of data collection was justified and described in detail.  Partially = The method of data collection was justified but only partially described.  No = The method of data collection was either not justified, not described, or both. | Yes = 2  Partially = 1  No = 0 |
|  |  |  |
| Q7. Was the qualitative data analysis sufficiently rigorous? | Yes = The method of data analysis used was appropriate for the research design and question.  Partially = Some statistical tests used were appropriate for the research design and question.  No = Statistical tests used were not appropriate for the research design and question. | Yes = 2  Partially = 1  No = 0 |
| **Quality Score Quantitative or Qualitative: /10** | | |
| **Quality Score Mixed Method: /14** | | |

Table S2. Quality Assessment Scores

| Study | Q1 | Q2 | Q3 | Q4 | Q5 | Q6 | Q7 | Total |
| --- | --- | --- | --- | --- | --- | --- | --- | --- |
| **Community** | | | | | | | | |
| Beier et al., 2021 | 2 | 2 | 2 | 0 | 0 | - | - | 6 / 10 |
| Bernard, 1975 | 0 | 1 | 0 | 0 | 0 | - | - | 1 / 10 |
| Cacciatori, 2017 | 2 | 2 | 1 | - | - | 2 | 1 | 8 / 10 |
| Dombert et al., 2016 | 2 | 2 | 2 | 0 | 1 | - | - | 7 / 10 |
| Dymond & Duff, 2020 | 2 | 2 | 1 | - | - | 2 | 2 | 9 / 10 |
| Extein, 2005 | 0 | 0 | 0 | - | - | 2 | 1 | 3 / 10 |
| Freimond, 2013 | 2 | 2 | 0 | - | - | 2 | 2 | 8 / 10 |
| Houtepen et al., 2016 | 2 | 2 | 1 | - | - | 2 | 0 | 7 / 10 |
| Ingram et al., 2024 | 2 | 2 | 1 | - | - | 2 | 2 | 9 / 10 |
| Jahnke et al., 2015 | 2 | 2 | 1 | 1 | 2 | - | - | 8 / 10 |
| Jahnke, Blagden et al., 2023 | 2 | 2 | 1 | 2 | 2 | 2 | 2 | 13 / 14 |
| Jimenez-Arista & Reid, 2022 | 2 | 2 | 1 | - | - | 1 | 1 | 7 / 10 |
| Lievesley et al., 2020 | 2 | 2 | 1 | 1 | 2 | - | - | 8 / 10 |
| Lievesley et al., 2022 | 2 | 1 | 0 | 1 | 2 | - | - | 6 / 10 |
| Mitchell & Galupo, 2018 | 0 | 2 | 1 | 1 | 0 | 1 | 1 | 6 / 14 |
| Morris, 2023 | 0 | 2 | 2 | - | - | 2 | 2 | 8 / 10 |
| Moss et al., 2021 | 2 | 2 | 1 | 2 | 2 | - | - | 9 / 10 |
| Pedersen, 2023 | 2 | 2 | 0 | - | - | 2 | 1 | 7 / 10 |
| Roche, 2020 | 2 | 2 | 1 | - | - | 2 | 0 | 7 / 10 |
| Roche et al., 2022 | 0 | 2 | 1 | 0 | 0 | - | - | 3 / 10 |
| Schaefer et al., 2022 | 2 | 2 | 1 | - | - | 1 | 0 | 6 / 10 |
| Shields et al., 2020 | 0 | 2 | 1 | - | - | 2 | 1 | 6 / 10 |
| Stephens & McPhail, 2019 | 2 | 2 | 1 | 1 | 0 | - | - | 6 / 10 |
| Stevens & Wood, 2019 | 2 | 2 | 0 | - | - | 2 | 1 | 7 / 10 |
| Tozdan et al., 2022 | 0 | 2 | 1 | 0 | 0 | - | - | 3 / 10 |
| Tozdan et al., 2023 | 2 | 2 | 1 | - | - | 2 | 1 | 8 / 10 |
| Walker, 2017 | 2 | 2 | 1 | - | - | 2 | 2 | 9 / 10 |
| Wilpert & Jansen, 2020 | 1 | 2 | 2 | 1 | 0 | - | - | 6 / 10 |
| **Clinical** | | | | | | | | |
| Beier et al., 2009 | 2 | 2 | 2 | 1 | 0 | - | - | 7 / 10 |
| Landgren et al., 2020 | 0 | 2 | 2 | - | - | 2 | 2 | 8 / 10 |
| Schaefer et al., 2010 | 0 | 2 | 2 | 0 | 0 | - | - | 4 / 10 |
| Stelzmann et al., 2022 | 2 | 2 | 2 | - | - | 2 | 1 | 9 / 10 |
| Wagner et al., 2016 | 2 | 2 | 2 | - | - | 1 | 0 | 7 / 10 |
| **Forensic** | | | | | | | | |
| Blagden et al., 2018 | 2 | 2 | 0 | - | - | 2 | 2 | 8 / 10 |
| Drapeau, Caspar et al., 2005 | 2 | 1 | 0 | - | - | 2 | 2 | 7 / 10 |
| Drapeau et al., 2005 | 2 | 2 | 0 | - | - | 2 | 1 | 7 / 10 |
| Walton & Duff, 2017 | 0 | 2 | 2 | - | - | 2 | 2 | 8 / 10 |
| **Mixed** | | | | | | | | |
| Boons et al., 2021 | 2 | 2 | 0 | - | - | 2 | 0 | 6 / 10 |
| Tozdan & Briken, 2019 | 0 | 2 | 2 | 0 | 2 | - | - | 6 / 10 |
| Vogt, 2006 | 2 | 2 | 1 | 2 | 2 | 1 | 0 | 10 / 14 |

Important Notes

- The quality analysis was developed strictly for the context of the current review and does not reflect the overall quality of a paper in the context of its own aims. For instance, if a paper has a good research question, but it is not related to the treatment needs and experiences of pedohebephiles, it will receive a score of 0 for Q1.
- Similarly, in such papers, we only assessed the quality of the items that were essential to the current review. For example, if a study has numerous valid and reliable measures, but the only measure that was of interest for the purpose of this review was one-item and/or unvalidated, the authors will have given it a rating of 0.
- Q4 and Q5 are applicable to quantitative studies; Q6 and Q7 are applicable to qualitative studies. Q4-Q7 are applicable to mixed methods studies. Therefore, quantitative-only and qualitative-only studies will have a total possible score of 10, whereas mixed methods studies will have a total possible score of 14.

Data Extraction Template

1. General Information
2. Country
3. Type of Study (online, in-person, document analysis, etc)
4. Methods
5. Research interest

- Treatment needs related to sexual interest and motivation to seek treatment.
- Prevalence of pedohebephiles wanting treatment.
- Treatment needs related to their sexual interest. This can include depression, anxiety, suicidal ideation or behaviours.
- Motivation to seek treatment. This can include avoiding stigmatization, meeting societal norms, and wanting to change their sexuality.
- Experiences seeking and receiving treatment.
- Treatment-seeking behaviors. This can include what they do to seek treatment such as reaching out to a therapist, or finding online support.
- Experiences in treatment. Only include if the pedohebephiles in question have been in treatment.
- Barriers and facilitating factors to treatment.
- Other:_______________________________

1. Aim of study: ____________________________
2. Study Design

- Quantitative
- Qualitative

1. Materials: ____________________________
2. Inclusion criteria: ____________________________
3. Exclusion criteria: ____________________________
4. Sample
5. Sample Size (Include only number of pedohebephilic participants): _________
6. Sample description

- Clinical
- Community
- Forensic
- Other

1. Assessment of attraction to children

- Self-reported
- DSM-V
- Penile Plethysmography
- Other

1. Exclusivity of Sexual Interest: ____________________________
2. Offense Status of Participants

- Offenders
- Non-offenders
- Unknown

1. Results
2. Age

|  | Mean/Median/Range, etc. |
| --- | --- |
| Age |  |

1. Gender

|  | N or % |
| --- | --- |
| Male |  |
| Female |  |
| Other |  |

1. Sexual Interest

|  | N or % |
| --- | --- |
| Nepiophilia |  |
| Pedophilia |  |
| Hebephilia |  |

1. Sexual Orientation

|  | N or % |
| --- | --- |
| Females |  |
| Males |  |
| Both |  |

1. Information on prevalence of pedohebephiles wanting treatment: _________________
2. Information on treatment needs related to their sexual interest: _________________
3. Information on motivation to seek treatment: _________________
4. Information on treatment-seeking behaviors: _________________
5. Information on experiences in treatment: _________________
6. Information on barriers and facilitating factors to treatment: ________________
